# Barriers faced by surgeons in identifying and managing malnutrition in emergency general surgery: A qualitative study

**DOI:** 10.1111/codi.17261

**Published:** 2024-12-10

**Authors:** Daniel L. Ashmore, Daniel M. Baker, Timothy R. Wilson, Vanessa Halliday, Matthew J. Lee

**Affiliations:** ^1^ School of Medicine and Population Health, Faculty of Health University of Sheffield Sheffield UK; ^2^ Department of General Surgery Doncaster and Bassetlaw Teaching Hospitals NHS Foundation Trust Doncaster UK; ^3^ Department of General Surgery, St James's University Hospital Leeds Teaching Hospitals NHS Trust Leeds UK; ^4^ Institute of Applied Health Research, College of Medical and Dental Sciences University of Birmingham Birmingham UK

**Keywords:** emergency general surgery, malnutrition, nutrition, qualitative study

## Abstract

**Aim:**

Many patients undergoing emergency surgery are malnourished. Identifying malnutrition is a prerequisite to offering targeted nutritional support. Guidelines exist but little is known regarding exactly how surgeons identify malnutrition, or the barriers that influence surgeons' clinical decision‐making. The aim of this work was to explore how consultant surgeons identify malnutrition in emergency general surgery (EGS) patients and the barriers to nutritional assessment and intervention.

**Method:**

Consultant surgeons with emergency surgery duties were invited to participate. Semi‐structured interviews were conducted online, audiovisually recorded and transcribed. An inductive approach was used for data analysis using the framework method. Coding and analysis were performed by two independent researchers using NVivo software. Themes were developed and reviewed with the supervising team. Interviews continued until data saturation was reached. Ethical approval was gained prior to interviews.

**Results:**

Eighteen interviews were conducted across three hospital settings. Identification of malnutrition consisted of three themes: ‘The surgeon’ (knowledge, experience, planning ahead); ‘The patient’ (selection, composition, clinical progress, operative considerations); and ‘The institution’ (collaboration, extended surgical team). Three themes encompassed barriers experienced: ‘The surgeon’ (understanding, culture, ownership, time constraints); ‘The institution’ (provision, staffing, conflict, hospital setting); and ‘The wider context’ (research, external factors). These influenced clinical decision‐making, which had two themes: ‘To join or not to join’ (risk taking, site of anastomosis) and ‘Nutritional support’ (timing, referral pathways).

**Conclusions:**

The identification and management of malnutrition in EGS is fraught with barriers, impacting operative and clinical decision‐making. Improvements in surgeon education, culture, collaborative working and resources are needed.

**What does this paper add to the literature?**

Many patients undergoing emergency general surgery (EGS) are malnourished. This qualitative study of consultant general surgeons demonstrates a varied approach and several barriers to the identification and management of malnourished EGS patients. This impacts operative and clinical decision‐making. Three areas requiring intervention to improve the nutritional management are discussed.

## INTRODUCTION

Malnourished patients have worse surgical outcomes [1, 2, 3] than well‐nourished patients. As many as 60% of patients who undergo emergency general surgery (EGS) are malnourished [1, 4]. No consensus definition of malnutrition exists, with a recent systematic review demonstrating a wide variety of methods used in the EGS literature [5].

Identifying and managing malnourished EGS patients is a dynamic process. Clinical guidelines exist with a range of screening tools, assessment processes and recommendations [6, 7, 8, 9, 10]. However, challenges persist such that the commencement of nutritional support is delayed [11, 12]. This ‘delay’ may reflect a complex decision‐making process as a result of several barriers encountered by surgeons [13]. This study explored this theory further with our aims being to (1) understand how consultant surgeons identify malnutrition, (2) explore the barriers that exist and (3) identify whether this practice is reflected across different hospital settings.

## METHOD

### Overview of study design

The design and reporting of this explorative qualitative study is in accordance with guidelines [14]. Written, informed consent was obtained from participants. Ethical approval was received from the University of Sheffield, UK (UREC 050436).

### Research team and reflexivity

Interviews were carried out by DA (PhD student/surgical trainee) following formal training. A summary of the research team's role and experience is provided in Table S1. Reflexivity has many definitions [15], and was addressed in several ways throughout this study (Table S2).

### Methodological approach

Wide variation in practice and several contextual barriers hinder the identification of malnutrition in EGS patients by senior decision‐makers [13]. However, there is no research exploring why this is so, or the presence and reason for other barriers that may also exist in complex healthcare systems. An exploratory approach with semi‐structured interviews was chosen to answer these questions with senior surgical decision‐makers.

An inductive approach was used for data analysis using the framework method, a type of thematic analysis [16, 17]. This approach is widely used in healthcare research and adopts a systematic process. Semi‐structured interviews allow researchers to explore ideas with deeper questioning while guiding the interview along a common framework. It can help draw conclusions around themes according to individuals or specific groups or organizations [17], including different hospital settings as in this study.

### Participant selection

Eligible participants were UK consultant surgeons who undertook EGS. Participants were purposively selected to ensure broad representation of EGS experience/practice with respect to sex, years qualified and hospital setting. These included district general hospitals (DGH), teaching hospitals (TH) and intestinal failure units (IFU). Forty‐eight EGS surgeons were contacted directly having registered an interest in previous studies conducted by the research team, of which nine responded and were interviewed. Additional recruitment was via social media platforms to EGS‐specific groups and relevant clinical speciality associations. Potential participants who had expressed interest were given the participant information sheet and interviews arranged for those willing to take part at a mutually convenient time. Electronic signed consent with verbal confirmation at the start of the interview was obtained.

### Data collection

Interviews were conducted online via the Google Meet™ video conference system (Google, Palo Alto, CA) using the predeveloped interview schedule (Supplementary Material). Only DA and the participant were present. The interview schedule was prepared by DA, ML and VH. Interviews began with a background to the study, after which open‐ended questions were used to build rapport. Descriptors of participant years qualified, current hospital setting and previous intestinal failure (IF) experience were recorded. The process of delivering nutrition was avoided unless this had subsequently changed their practice in how they identify malnutrition. Additional questions were asked as needed or to follow up important points. Interviews were audiovisually recorded, performed once only and were anticipated to last no longer than an hour. Supporting field notes were taken and informed interview schedule development to explore emerging themes and issues related to reflexivity.

### Data analysis and coding

Interviews were transcribed by DA. Transcripts were not returned to participants. Themes were identified using well‐described methods [16]. Coding was undertaken by DA for all transcripts using NVIVO v.11 [18]. Half of transcripts underwent dual review and coding, with development of a thematic framework between DA and DB and agreement from the research team. Participants did not provide feedback on the findings. Data saturation was reviewed following 12 interviews, after which it has previously been shown to occur [19]. ‘Meaning saturation’ can require at least 16 interviews [20]. Interviews were performed until data saturation with no new themes identified.

## RESULTS

Eighteen interviews were conducted across three hospital settings. All surgeons worked in different units, except for the four IFU surgeons who worked across two organizations. Fourteen surgeons were male. Surgeon experience ranged from 6 months to 23 years (mean 7.5 years). Mean interview length was 44 min (range 26–58 min). A summary of participant characteristics is provided in Table 1.

**TABLE 1:** Participant characteristics.

Participant	Sex	Years as a consultant	Hospital setting	Experience with intestinal failure	Primary clinical subspeciality
1	M	10.5	Teaching	Yes	UGI OG
2	F	5	Teaching	No	EGS and major trauma
3	M	10	DGH	No	EGS
4	M	2.5	Teaching	No	EGS
5	M	6.5	Teaching	No	EGS
6	M	13	Teaching	Yes	Colorectal
7	F	4	Teaching	No	EGS and major trauma
8	M	4	Teaching	No	Colorectal and EGS
9	M	9	Teaching	Yes	HPB and EGS
10	F	0.5	Teaching	No	Colorectal
11	M	17	IF unit 1^a^	Yes	Colorectal and IF
12	M	2.5	Teaching	No	UGI and bariatrics
13	M	6	DGH	No	UGI
14	M	4	IF unit 1^a^	Yes	Colorectal and IF
15	M	23	DGH	No	UGI
16	M	4	IF unit 2^b^	Yes	Colorectal and IF
17	F	3.5	IF unit 2^b^	Yes	Colorectal and EGS
18	M	10	DGH	No	Colorectal

*Note*: Experience with IF includes time as a trainee/fellow roles. Superscript a and b indicate participants from the same hospital setting.

Abbreviations: DGH, district general hospital; EGS, emergency general surgery; F, female; IF, intestinal failure; M, male; OG, oesophago‐gastric; UGI, upper gastrointestinal.

**FIGURE 1:**
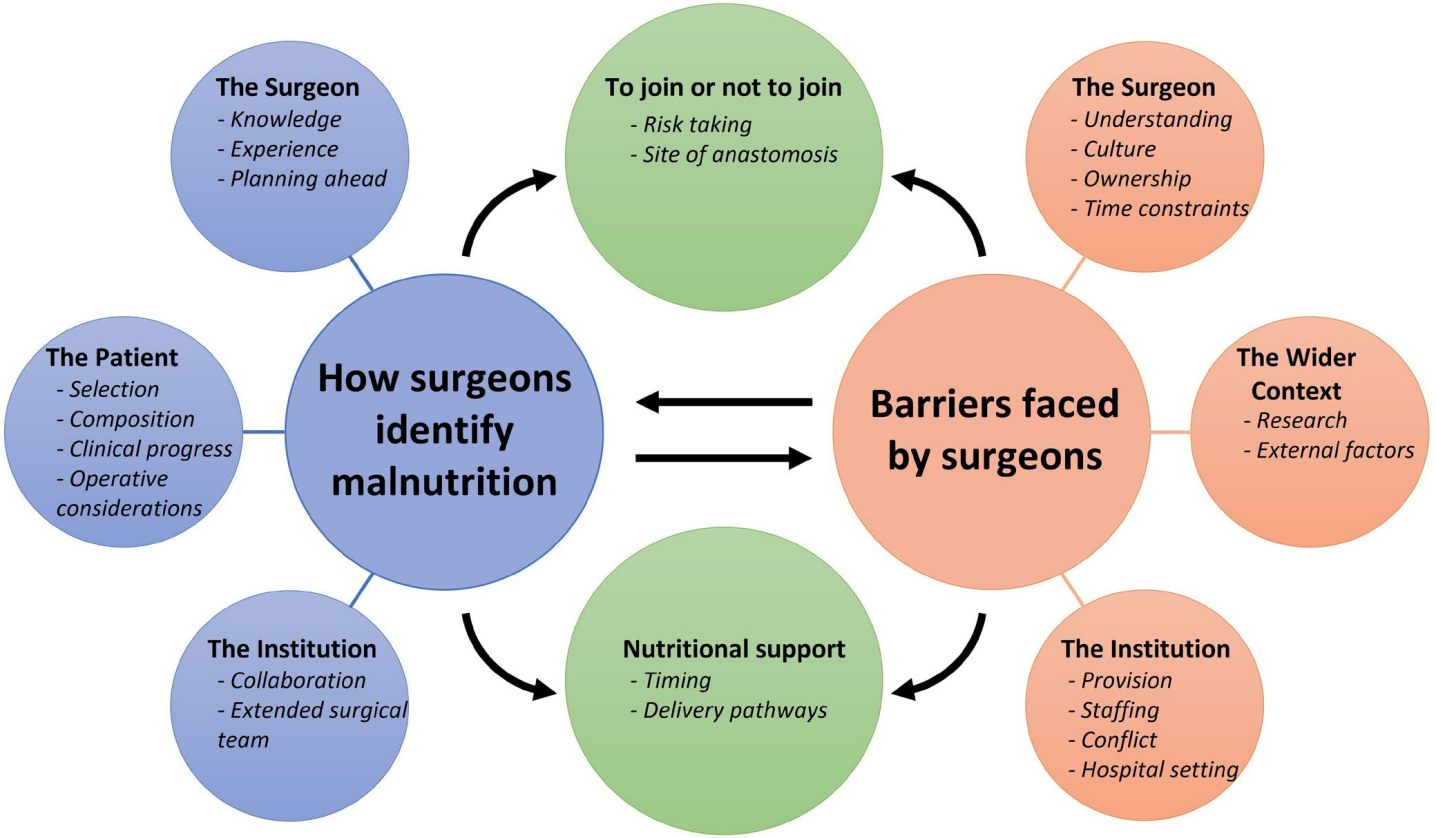
Themes and subthemes identified: how surgeons identify malnutrition (blue); barriers faced by surgeons (red); emerging themes/subthemes (green).

Themes identified and data saturation are presented in Figure 1 and Table 2. Analysis of the data relating to the identification of malnutrition revealed three overarching themes: ‘The surgeon’, ‘The patient’ and ‘The institution’ (Table 3). The data pertaining to barriers also yielded three themes: ‘The surgeon’, ‘The institution’ and ‘The wider context’ (Table 4). These influenced clinical decision‐making. It had two themes: ‘To join or not to join’ and ‘Nutritional support’ (Table 5).

**TABLE 2:** Summary of themes with data saturation of subthemes.

Themes	Subthemes	Number of participants referencing subtheme, *n* (%)
How surgeons identify malnutrition		
The surgeon	Knowledge	18 (100.0)
	Experience	14 (77.8)
	Planning ahead	7 (38.9)
The patient	Selection	18 (100.0)
	Composition	17 (94.4)
	Clinical progress	4 (22.2)
	Operative strategy	18 (100.0)
The institution	Collaboration	12 (66.7)
	Extended surgical team	5 (27.8)
Barriers experienced by surgeons		
The surgeon	Understanding	13 (72.2)
	Culture	12 (66.7)
	Ownership	14 (77.8)
	Time constraints	11 (61.1)
The institution	Provision	14 (77.8)
	Staffing	8 (44.4)
	Conflict	12 (66.7)
	Hospital setting	4 (22.2)
The wider context	Research	12 (66.7)
	External factors	9 (50.0)
Decision‐making		
To join or not to join	Risk taking	11 (61.1)
	Site of anastomosis	6 (33.3)
Nutritional support	Timing	10 (55.5)
	Delivery pathways	10 (55.5)

**TABLE 3:** Illustrative quotes related to how surgeons identify malnutrition.

Theme and subtheme	Supporting quotations
The surgeon	
Knowledge	P4 (TH): ‘I'm not sure it's taught particularly well or I've ever had any sort of formal training in assessing someone's nutrition status’ P7 (TH): ‘I think it might be somewhere from medical school time when it was indoctrinated somewhere, “Don't operate on people with whom albumin is less than 20 or 25.” You just don't touch them’
Experience	P1 (TH): ‘It's mainly about eyeballing them’ P10 (TH): ‘I had one where I regretted anastomosing her because she leaked after a right hemi. It could have been multifactorial … it could have been a variety of comorbidities, but [she] was nutritionally poor’ P18 (DGH): ‘You've gotta be a bit careful with suntans and makeup but you get an idea of someone's nutritional status by looking at them’
Planning ahead	P6 (TH): ‘… what you're gonna do is you're gonna write down in your operation note, or at least I do, what my nutritional strategy is going to be for that individual patient tailored to what they've had done, what I think their nutritional risk were beforehand, mostly made up off a fag packet, and with the view to, you know, optimizing their recovery’ P10 (TH): ‘I don't think you can really predict it’ P14 (IFU): ‘I think it's common sense to us, whether it's common sense to everybody? But I think it is. Are they eating? No. When did they last eat? It was a week ago. Right, they've been picking for a week. When are they likely to go from picking to eating and get all their calories? If it's not anytime soon based on this trajectory, we will get them some TPN’
The patient	
Selection	P3 (DGH): ‘Somebody who's 20 stone but has managed to lose 3 stone, very rapidly, is almost more concerning sometimes than somebody who's at the lower end and who's only lost a couple of pounds’ P6 (TH): ‘But I think the MUST score's probably of limited use … I don't know of a robust validated scoring system for nutrition in the acute setting’ P15 (DGH): ‘For me, the history is 3 days or more patients not being fed with features of vomiting, diarrhoea, small bowel obstruction with peritonitis … I immediately ask them to be post‐op put on TPN’ P18 (DGH): ‘Why can't this person just eat and drink as normal?’
Composition	Albumin P1 (TH): ‘I mean, you know, albumin is a very poor marker of nutrition. It's very late one’ P2 (TH): ‘I find myself using [albumin] … when I said “This patient needs TPN because their albumin's 12”. They're [dietitians] like, “No, their albumin's 12 because they're septic. That's not a good reason to give it” ’ P7 (TH): ‘My personal level that I use a lot is albumin, just as simple as that. It's all on a blood test and you can fairly quickly see if there is a problem or not’ P13 (DGH): ‘I would probably not be embarrassing enough to phone up a dietitian and go, “I'd like you to put TPN on this patient because their albumin is like 25,” [laughing] … [they will] just probably making fun of me!’ P18 (DGH): ‘And people talk a lot of nonsense about nutrition. They just don't know what they're talking about … They'll go, “The albumin's okay. They're not malnourished” ’ Body mass index P2 (TH): ‘… obese patients can lose a lot of weight without looking thin and their malnutrition could be missed’ P5 (TH): ‘So, the idea of the very, very overweight, but also malnourished patients, which I know is an absolute fundamental of dietitian practice and all the rest of it, I would say I'm not a massive believer in’ P14 (IFU) ‘But I think from a nutrition point of view, rightly or wrongly, we don't worry about the fat ones so much … probably because we don't understand it isn't it? We can see unhealth and think it's skinny people and frailty … there's a philosophical thing around culture and increasingly high BMI being the normal’
Clinical progress	P4 (TH): ‘Is the patient still in ileus you know? Are things settling down? That sort of thing as a guide to the longevity of pathology, with a view to then guiding their nutrition’
Operative considerations	P14 (IFU): ‘Yeah, but the devil's in the detail isn't it?’ P17 (IFU): ‘What are the consequences of not operating because it's not a zero‐sum game’
The institution	
Collaboration	P6 (TH): ‘I think you've got to trust others – their expertise’ P9 (TH): ‘So the nutrition team help us by sort of, it's like educating us, you know?’ P10 (TH): ‘It's something that I very much need some guidance and some help with’ P14 (IFU): ‘… he actually finds it much more difficult than I do to access services for these patients … he just doesn't have a day‐to‐day working relationship with them and sharing patients with them all the time’
Extended surgical team	P2 (TH): ‘I think we've got very good dietitian services for the ward … it's got an intestinal failure MDT that meets once a week to discuss the patients on TPN’ P11 (IFU): ‘We've got the specialist team that are involved with IF patients on the unit … but we recognized the patients on the surgical ward who were not under the IF team got a pretty, you know, second class service’

Abbreviations: DGH, district general hospital; IF, intestinal failure; IFU, intestinal failure unit; MUST, Malnutrition Universal Screening Tool; TH, teaching hospital; TPN, total parental nutrition.

**TABLE 4:** Illustrative quotes related to barriers encountered by surgeons.

Theme and subtheme	Supporting quotations
The surgeon	
Understanding	Lack of understanding P3 (DGH): ‘I don't think there's any barriers in me making the decision other than my own ignorance’ P7 (TH): ‘If you don't know there is a problem, you can't fix a problem’ P9 (TH): ‘I appreciate nutrition is important … but I didn't realize how important and also, I didn't really appreciate why’ P10 (TH): ‘Certainly [patients] don't understand the implications of it in the context of emergency surgery because I don't think we understand it that well to be honest’ P12 (TH): ‘It's just yet another thing that's been focused on within enhanced recovery and things in elective patients, where the emergency care's been a bit left behind is now coming to the forefront’ P16 (IFU): ‘… there was this lack of awareness that when they come as an admission, or their index presentation, is not when their symptoms have started. They're already on this kind of decline, so to say nutritionally’ Improving understanding P3 (DGH): ‘… if there was like a little nutrition teaching package for surgeons … would be good’ P9 (TH): ‘Empowerment … it was the empowerment of the surgeon to influence nutrition … how dare you, as a surgeon, even start talking to the nutrition team about nutrition. It was like, you know, I had absolutely no say in nutrition. And I'd just grown up with that, you know’ P10 (TH): ‘I think there could do with being more surgical representation’ P11 (IFU): ‘I know there's some stuff in ISCP [surgical training portfolio] but it's pretty vague’ P14 (IFU): ‘The message being brought from the dietetic … to something you're hearing about at ASGBI and ACPGBI [major UK surgical societies]’ P17 (IFU): ‘… now that you have the rise of emergency surgery as a specialty, it's actually a really exciting’
Culture	P1 (TH): ‘But there's still a lot of dogma in surgery, you know. There's still a lot of old practices, you know’ P11 (DGH): ‘Surgeons want to “surge” ’ P12 (TH): ‘… nutrition and malnutrition is one of many aspects of care that is kind of “non‐surgical” in that it doesn't involve the operation’ P13 (DGH): ‘It's sort of an old‐fashioned sort of way of talking. It's like an old language, because everyone did believe that albumin was the one and only marker of nutrition’ P14 (IFU): ‘My unit is very internally appointed … therefore their behaviours and practices all mirror one another’
Ownership	P1 (TH): ‘So if I if I decide somebody needs TPN I have to refer to the nutrition team. I think it's in some ways it takes the pressure of the decision off you a little bit because it's not something I am an expert at by any stretch the imagination and it's something that I very much need some guidance and some help with, and that's what they do really, really well’ P6 (TH): ‘Usually in my experience, if I'm the surgeon in charge of their care, they [the nutrition team] will usually go along with what I say unless they think something's not safe from an airway perspective’ P8 (TH): ‘So if I've got a patient that I think needs nutrition, the barriers we come up against are sometimes there can be a delay to assessment and an authorization … pharmacy will then sometimes recontact the ward and say, “Well, that's fine. We can issue that. But only once we've had authorization from the nutrition team” ’ P9 (TH): ‘It's my name above the bed’ P9 (TH): ‘[Gastroenterologists] used to come along and say, “This is the nutrition plan.” I'd say, “I disagree with it so therefore you need to take the patient over.” They would say, “Well we're not the named consultant therefore, there you go.” I'd go, “Great. In which case I want TPN” ’ P15 (DGH): ‘I think like everything in the NHS or in the healthcare [nutrition decisions] should be MDT approach. And even by calling them it's becoming an MDT approach’ P17 (IFU): ‘It's shared decision‐making between the nutrition team and the surgeon responsible for the patient and often in the emergency setting, the intensivists’ P18 (DGH): [If the participant disagrees with the nutrition team's assessment] ‘Well look, I'm gonna override that because I'm in charge.’ … I don't think [surgeons] can do any of this work … Expecting us to assess and do all this stuff as well as other people is just nuts. It's just old‐fashioned thinking’
Time constraints	P1 (TH): ‘We don't have that luxury in emergency surgery. You know, time's limited’ P6 (TH): ‘I think if the impact of poor nutritional management was better known, if there is a significant one – I suspect there will be – and there's some data out there, then I think it would go up people's priorities and people will be more inclined to make time for it’ P15 (DGH): ‘Can you improve the nutrition within 12 hours? [You're] unlikely to make them much fitter’ P6 (TH): ‘We've got so many things to focus on … nutrition's probably further down the pecking order at the minute because we know so little about it’ P16 (IFU): ‘… a proper nutritional assessment takes an hour at the very least…you just don't have that’
The institution	
Provision	P7 (TH): ‘… a lot of things depends on as well which time of the day we are and what day of the week it is because if it's Friday afternoon, you know you're not gonna get anything. It's just because nutritional team is already gone. To get TPN, it's a nightmare. We can generally get emergency ones because we have, every ward has emergency supplies, so we can use that in the worst‐case scenario’ P12 (TH): ‘Absence of 7‐day working is another problem’ P15 (DGH): ‘The worst cases are when they come Friday afternoon. Because you have Saturday and Sunday ahead. And the nutrition team is unlikely to be available. And if it is a Bank Holiday Monday, it's even worse that it can go up to Tuesday. And until they see them on Tuesday, they can't start TPN until Wednesday … and you almost lose a week’ P17 (IFU): ‘[On reflecting on challenges working in a non‐IFU] … availability of TPN, comfort around it, nursing, logistics of being able to give TPN, being able to manage the lines, recurrent line sepsis, availability of a line service …’ P18 (DGH): ‘… trying to get a line put in to have some TPN is a nightmare’
Staffing	P3 (DGH): ‘But on other wards where there may be two nurses to 33 patients. They're lucky if they can get some food … the support that sometimes people need in eating, in physically getting the food to their mouths, we seem to struggle to do now because we don't have enough staff to do it’ P11 (IFU): [In relation to PICC line infections and thromboses] ‘… it turned out that junior doctors were taking blood from the lines and nobody told them that they shouldn't’ P18 (DGH): ‘There's a permanent staffing crisis’
Conflict	P5 (TH): ‘So I think we would we would ask for it a lot earlier; it's just that they [gastroenterologists] tend to say no if it's not going to be sort of 7 days or more’ P6 (TH): ‘… getting a unified position, can sometimes be challenging’ P9 (TH): ‘… it was the empowerment of the surgeon to influence nutrition … you dare not, how dare you as a surgeon, even start talking to the nutrition team about nutrition’ P13 (DGH): ‘… we’re carrying the can for this decision and so if I think they need TPN, ultimately that should be the case … If it's all just opinion‐based then my opinion wins’ P14 (IFU): ‘I don't think I've had a request for TPN challenged’
Hospital setting	P11 (IFU): ‘… there's no doubt that we do things here that we know we can because we do it in other patients’ P14 (IFU): ‘I think we probably are [too liberal with nutritional support]. I'm sure there are patients where we use it for 3, 4 or 5 days … in any other hospital, that's just you know, that's rocking horse poo’ P16 (IFU): ‘The fact that I work in an IF unit … we'll deal with it’ P17 (IFU): ‘So we know that we've got ease of access to TPN etc so we would be less likely to do an anastomosis in the context where we would, sort of, be doing it on a hope and a prayer’ P18 (DGH): ‘You have to work with what you've got. You learn that very early on … there are lots of people that work in privileged institutions that have a privileged role … but if you're jobbing away as a jobbing general surgeon, you have to work with what you've got’
The wider context	
Research	P1 (TH): ‘… if a surgeon doesn't agree with it, they'll just pick holes in it and not believe in it … even with a well, you know, a well‐studied randomized trial’ P9 (TH): ‘I really, really think that nutrition in the surgical patient should be talked about at AUGIS, ASGBI and ACPGBI [major UK surgical societies]. But it needs to be chalked in a way that surgeons understand’ P11 (IFU): ‘I'm stuck because I genuinely don't know how you would improve all of that. And you know, I mean there are guidelines as we have described from ESPEN, from NICE, but you know the trouble is with guidelines is that you've got to read them to understand them, you know. And people don't find time to do that, or because it's not necessary’ P13 (DGH): ‘What we don't have I think is a very good understanding of what happens afterwards; what's their recovery like, what's the difference? P14 (IFU): ‘You'll never get ethics to starve half your EGS population and feed the other half’
External factors	P1 (TH): ‘… the hospital food is maybe not what they require … it's done very cheaply’ P3 (DGH): ‘Even the quality of the hospital food that people get after they've had their appendix out. You know, it's unlikely to compromise them … I'm not sure that's particularly high on the whole NHS radar’ P6 (TH): ‘I think if you looked at a postcode of where your patients are from, you're probably looking at fairly different nutritional parameters based on your postcode just like many other things are’ P16 (IFU): ‘… NICE maybe needs to take this on … and say it becomes maybe a key performance indicator’

Abbreviations: ACPGBI, Association of Coloproctology of Great Britain and Ireland; ASGBI, Association of Surgeons of Great Britain and Ireland; AUGIS, Association of Upper Gastrointestinal Surgery of Great Britain and Ireland; DGH, district general hospital; EGS, emergency general surgery; IF, intestinal failure; IFU, intestinal failure unit; ISCP, Intercollegiate Surgical Curriculum Programme; MDT, multidisciplinary team; NHS, National Health Service; NICE, National Institute for Health and Care Excellence; PICC, peripherally inserted central catheter; TH, teaching hospital; TPN, total parenteral nutrition.

**TABLE 5:** Illustrative quotes related to clinical decision‐making by surgeons.

Theme and subtheme	Supporting quotations
To join or not to join	
Risk taking	P1 (TH): ‘It might change what I do at the operation. It might sway me to not do an anastomosis. But that would be a part of the global picture. It wouldn't be not doing any anastomosis just because they're malnourished’ P13 (DGH): ‘It appears that nowadays a more conservative approach that avoids an anastomosis, and as such avoid the risk of a leak, and the idea is survival, and maximizing survival is to give you a stoma … If they're malnourished it's just another reason not to join them up’ P14 (IFU): ‘I think the decision of to join or not to join is probably more from other factors than just malnutrition … Malnutrition is just another tick in that box’
Site of anastomosis	P9 (TH): ‘[in the context of hypoalbuminaemia] … you are not going to be doing an anastomosis in that patient and if the problem's in the small bowel, for example, you may end up with a high‐output ileostomy and therefore you're going to compound the already poor nutritional state that that patient is likely in’ P17 (IFU): ‘So we know that we've got ease of access to TPN etc so we would be less likely to do an anastomosis in the context where we would, sort of, be doing it on a hope and a prayer … just because it's a relatively proximal piece of bowel, we have little compunction to taking out a relatively proximal piece of bowel [forming a stoma] rather than doing anastomosis’
Nutritional support	
Timing	P3 (DGH): ‘So, you know, if we'd have just ploughed in and done surgery on him, he'd have just fallen apart and died, I'm sure. And it's sometimes that bit of knowing when not to operate and go, “Right. Yeah. This all looks really horrible” but actually recognizing that you're so nutritionally deplete that if we try and do something, it isn't going to work’ P7 (TH): ‘Do I need to get them any parenteral nutrition now or will I have to do it after a day or two? Or like, do I have to start it before surgery? Or, which takes a priority? Do they need surgery first and then try to top them up? Or do I need to start it now before surgery and then to continue it afterwards?’ P10 (TH): ‘They tend to have a slightly smoother course, I think, if they're in a better shape beforehand’
Delivery pathways	P4 (TH): ‘It's very much, you know, if you want someone on TPN, you say, “Put them on TPN” ’ P18 (DGH): ‘… they're just more at risk than all the elective patients we tee up and investigate to the hilt beforehand … we sort of leave, comparatively, all these other patients to just get on with it because well, “They're ill so it's fine”. … But we need to sort of reverse that mentality and be all over them because we could actually probably make a bigger difference to them’

Abbreviations: DGH, district general hospital; IFU, intestinal failure unit; TH, teaching hospital; TPN, total parental nutrition.

This manuscript specifically presents results focusing on the barriers experienced by surgeons; however, illustrative quotes are provided for all themes and subthemes (Tables 3, 4, 5). We then draw together all themes in our discussion, identifying areas that may offer the greatest opportunity to implement change and improve outcomes for patients.

### Barriers experienced by surgeons

#### The surgeon

Four subordinate themes were identified which related to the surgeon. These were ‘Understanding’, ‘Culture’, ‘Ownership’ and ‘Time constraints’.

#### Understanding

Surgeons admitted a lack of understanding in various areas. These pertained to the importance of nutritional support in EGS, difficulty weighing the risks and benefits of nutritional support, limited awareness that being malnourished is important and ignorance across all these levels. Opportunities to improve understanding centred on teaching packages, empowerment, representation in nutrition multidisciplinary teams (MDTs) and dissemination of learning at surgical conferences.

I don't think there's any barriers in me making the decision other than my own ignorance. P3 (DGH)

#### Culture

Surgical nutrition was viewed as a ‘nonsurgical’ component of surgical care, rife with dogma and old traditions that are perpetuated across generations. For some, an operation was considered paramount in the cure of patients' problems. Albumin is still considered a common language between surgeons due to historic misconceptions that it is diagnostic of malnutrition.

Surgeons want to ‘surge’. P11 (DGH)

It's like an old language, because everyone did believe that albumin was the one and only marker of nutrition. P13 (DGH)

#### Ownership

Many surgeons were ingrained in the belief that they are solely accountable for the patient. However, other surgeons were accepting of guidance from other healthcare professionals and a recognition that identifying and managing nutrition may not fall within a surgeon's expertise.

It's my name above the bed. P9 (TH)

… expecting us to assess and do all this stuff as well as other people is just nuts. It's just old‐fashioned thinking. P18 (DGH)

#### Time constraints

Surgeons repeatedly raised the issue that they are busy clinicians. Two main issues arose: a lack of time and a need to prioritize. The former pertained to busy ward rounds, needing to learn how to formally diagnose malnutrition and often because the patient imminently needs an operation.

Can you improve the nutrition within 12 hours? [You're] unlikely to make them much fitter. P15 (DGH)

Surgeons discussed many conflicting priorities resulting in malnutrition being considered less important.

We've got so many things to focus on … nutrition's probably further down the pecking order at the minute because we know so little about it. P6 (TH)

### The institution

Again, four subordinate themes were identified and related to barriers as a result of the institution within which surgeons work: ‘Provision’, ‘Staffing’, ‘Conflict’ and ‘Hospital setting’.

#### Provision

While access to line services and nutritional support was considered adequate by some surgeons, many considered it poor, especially over the weekend when services were not available.

Absence of 7‐day working is another problem. P12 (TH)

#### Staffing

Staff vacancies were considered a major issue, in particular with regard to access to dietitians with appropriate experience. One surgeon explained that basic care and nutritional support has been affected.

… there may be two nurses to 33 patients. They're lucky if they can get some food. (P3 DGH)

#### Conflict

Conflict can arise regarding whether to give nutritional support to patients or not. While many surgeons discussed the value of collaborative working, several surgeons felt other team members were ‘gatekeepers’ to nutritional support. This was not an issue for surgeons in IFUs.

*…* getting a unified position, can sometimes be challenging. P6 (TH)

I don't think I've had a request for TPN [total parenteral nutrition] challenged. P14 (IFU)

#### Hospital setting

There were differences in the narrative between non‐IF units and IF units. Surgeons at IFUs described easy access and the presence of supporting teams able to manage nutrition. Additionally, their elective practice informed their EGS practice. Altogether, this appeared to change clinical decision‐making, such as having little concern for patients with high‐output stomas or patients who would likely need a protracted period of nutritional support.

The fact that I work in an IF unit … we'll deal with it. P16 (IFU)

… if you're jobbing away as a jobbing general surgeon, you have to work with what you've got. P18 (DGH)

### The wider context

Two subthemes related to the wider context: ‘Research’ and ‘External factors’.

#### Research

There was concern for a lack of evidence regarding nutritional support in the EGS setting, including long‐term outcomes for patients and resultant service implications. This appeared to lead to a reliance on experience and opinion. A number of surgeons commented on the quality of the current data and that surgeons wouldn't trust them in any case.

… if a surgeon doesn't agree with it, they'll just pick holes in it and not believe in it … P1 (TH)

Further, it was recognized that although standards do exist, surgeons need to have read them, understood them and then use them in their practice. It was expressed that dissemination of such guidelines needs to be surgeon friendly.

… it needs to be chalked in a way that surgeons understand. P9 (TH)

#### External factors

The finite financial resources available was a recurring issue. Surgeons described the funding of services being stretched such that basic nutrition is compromised. A minority of surgeons commented that the nutritional requirements of patients may vary depending on where they live, and that these differences can lead to a ‘postcode lottery’ effect.

… the hospital food is maybe not what they require … it's done very cheaply. P1 (TH)

## DISCUSSION

This study used qualitative methods to explore current practice and barriers to consultant surgeons in the identification and management of malnutrition in the emergency setting in the United Kingdom. We confirm variation in how surgeons identify malnutrition and report several contextual barriers that hinder this process. This corroborates findings from previous studies that have explored current methods for nutrition evaluation [2, 5, 13]. We draw attention to three areas that require intervention to improve the nutritional management of EGS patients.

### Surgeon understanding and culture

Surgeons recognized that their understanding about how to identify malnutrition was poor. Two approaches still in common use may result in misdiagnosis or missing malnutrition entirely. Firstly, many still place much emphasis on hypoalbuminaemia in diagnosis of malnutrition, drawn by its ‘objectivity’. It is still used as a *‘*language’ to convey the nutritional status of a patient—that a patient with hypoalbuminaemia is malnourished. Like malnutrition, hypoalbuminaemia is also prognostic of poor outcomes, including mortality [21, 22, 23]; but it is important to dissociate them since many patients with malnutrition have a normal albumin. Conversely, while there may be some correlation between hypoalbuminaemia and malnutrition—hypoalbuminaema is indicative of inflammation which carries a nutritional burden—patients with hypoalbuminaemia are not always malnourished and in need of nutritional support. The pathophysiology of hypoalbuminaemia has been clearly described [8, 24, 25]. Without severe prior malnutrition or in the presence of inflammatory disease, hypoalbuminaemia in the emergency setting does not have a nutritional component [24, 26]. Consequently, nutritional guidelines all advise that albumin should not be a marker of malnutrition acutely [6, 7, 8, 9, 10]. However, as one surgeon (P11) recognized, surgeons have ‘to read them to understand them … and people don't find time to do that, or because it's deemed not necessary’.

Secondly, surgeons described being able to diagnose malnutrition by ‘eyeballing’ patients. One IFU surgeon stated, ‘we don't worry about the fat ones so much’. Use of this ‘end of the bed assessment method’ is also recognized by surgeons when identifying high‐risk surgical patients [27]. While this approach may work for the overtly cachectic patient it is of little use in others. Obese patients may have micronutrient deficiencies and severe metabolic impairment that will have a deleterious impact on their operative outcome. Surgeons must recognize that it is the patient's hidden internal body processes that are of concern and not their outward appearance. This can only be uncovered by obtaining a nutrition history. Without this, malnutrition will be underrecognized and inappropriately treated [28, 29, 30].

The same surgeon recognized ‘a philosophical thing around culture and increasingly high BMI [body mass index] being the normal’. Others recognized dogma and traditions affecting their practice. Literature alludes to the existence of a surgical personality [31, 32, 33], norms [34] and culture [35, 36]. Nutrition in EGS was described as ‘nonsurgical’. Conscientious surgeons questioned not knowing when best to operate—before or after nutritional support if the diagnosis and time permitted. In an environment where the ‘behaviours and practices all mirror one another’, a change in surgical culture is needed. Significant changes in surgical training may help, as will disseminating the evidence and guidelines ‘chalked in a way that surgeons understand’. A change in surgical safety culture driven by implementing human factor strategies indicates that cultural shift is possible in the surgical environment [37, 38].

### Collaborative dialogue

Surgeons expressed feelings of ownership and duty towards patients. This sentiment has been described previously [34], and often relates to who makes the final decisions regarding a patient's care.

In the context of nutrition in EGS, some surgeons recognized they were not silos. Without the ‘luxury’ of time and a limited understanding, some surgeons eagerly collaborated with other healthcare professionals to build solid working relationships. Without these relationships, surgeons stated that other surgical colleagues found it ‘much more difficult than I do to access these services for these patients’. Value was seen in the ‘expertise’ offered by dietitians and an MDT approach.

However, conflict was described. Circumstances were related to a variety of issues: diagnosing malnutrition; indication and duration for nutritional support; likely clinical progress; what the expected *‘exit strategy’* was; and whether nutritional support would be of benefit at all. Rather than shared decision‐making, or deferring to the expertise of their colleagues, surgeons often cited they were ‘carrying the can’. In such scenarios, surgeons described asking dietitians to stop reviewing patients or even transferred patients to another centre, because ‘if it's all just opinion‐based then my opinion wins’. A range of healthcare professionals were viewed as gatekeepers to starting nutritional support, to the frustration of surgeons. This was not seen at IFUs, with one surgeon wondering if they were ‘too liberal with nutritional support’.

Shared ownership and a flat hierarchy have been suggested to avoid such problems [39, 40]. Other approaches may include surgical representation on nutrition MDTs to aid ‘negotiated settlements’.

### Adequate and equitable service provision

Finally, surgeons consistently recognized that finite resources within a healthcare system such as in the United Kingdom were a major barrier in EGS nutritional management. This was mainly inhibited by a ‘permanent staffing crisis’, a lack of staff experience and limited access to line and nutrition services. These issues have been described previously [41]. The cost of providing an EGS service is expected to increase in the United Kingdom [42] and these issues may worsen if funding is not increased accordingly.

Some surgeons battled to know what is best for patients, and this was influenced by the hospital setting they worked in. Clinical decisions, such as to perform an anastomosis or not, were occasionally grounded in whether nutritional support services were available locally. Nonclinical influences such as geography, socioeconomics and resource availability also affect clinical decision‐making [43, 44]. As well as having a life‐changing impact for patients, surgeons expressed regret following intraoperative decisions such as performing a risky anastomosis which resulted in a poor outcome. In contrast, surgeons working in IFUs felt they were supported adequately, and the availability of services did not affect their decision‐making. Their ‘privileged position’ meant they could simply ‘deal with’ the consequences. Wider issues such as the quality of hospital food and the differential nutritional baseline of patients across the country also exist.

Adequate investment is required to strengthen the provision for malnourished EGS patients so that decisions regarding nutritional support are founded on need rather than availability. This may ease the emotional decisional burden that surgeons experience.

### Strengths of the study

A strength of this study is in its methodology. There were planned postinterview debriefs; immersion in the data through transcription of interviews; dual coding and development of themes; and reporting in line with current guidelines [14]. Four of the 18 (22%) surgeons were female. A 2023 UK national consensus reported that just 17% of consultant general surgeons were female [45]. Additionally, a range of surgeon experience, subspeciality interest and hospital setting suggest that findings may be generalized to other healthcare settings. The qualitative approach provides granularity and an understanding of ‘why’ that may not be accessible through quantitative studies.

### Limitations of the study

There are potential limitations with this study. Self‐selection bias was minimized through broad recruitment of 18 surgeons across 16 centres. Surgeons may practice differently from how they described in interviews, but the conduct of the interviews should mitigate any pressure surgeons felt to offer ‘correct’ answers. The use of case vignettes for surgeons to reflect on, as used by some studies [34], was considered to be unhelpful in this instance. Nutrition is a vastly complex topic; ‘the devil is in the detail’, as one surgeon proffered. Experiences of other members of the nutrition team would be beneficial and may serve a potential area for future research.

## CONCLUSION

Several barriers affect the approach surgeons use to determine malnutrition in EGS patients, impacting clinical and operative decisions. This justifies changes in surgeon education, culture, collaborative working and improved resources and funding for EGS.

## AUTHOR CONTRIBUTIONS

**Daniel L. Ashmore:** Conceptualization; data curation; formal analysis; investigation; project administration; visualization; writing – review and editing; writing – original draft; methodology. **Daniel M. Baker:** Formal analysis; validation; writing – review and editing; visualization. **Timothy R. Wilson:** Supervision; writing – review and editing; visualization. **Vanessa Halliday:** Conceptualization; methodology; supervision; visualization; writing – review and editing. **Matthew J. Lee:** Conceptualization; methodology; visualization; writing – review and editing; supervision.

## FUNDING INFORMATION

The University of Sheffield Institutional Open Access Fund funded the article processing charges. There were no other sources of funding.

## CONFLICT OF INTEREST STATEMENT

The authors declare no conflicts of interest.

## ETHICS STATEMENT

Ethical approval was received from the University of Sheffield, UK (UREC 050436).

## PREVIOUS COMMUNICATION

This study is not based on any previous communication to a society or meeting. It was presented as an oral presentation to the Association of Surgeons in Great Britain and Ireland 2024 annual conference and the Association of Surgeons in Training 2024 annual conference.

## Supporting information


Table S1.

## Data Availability

Data can be made available on request.
