# Supplementary material for: Barriers faced by surgeons in identifying and managing malnutrition in emergency general surgery: A qualitative study
Source: Colorectal Dis. 2024 Dec 10;27(1):e17261. doi: 10.1111/codi.17261 (PMC11683319; doi:10.1111/codi.17261)
Supplement: Supplementary file 1 — Table S1. [file CODI-27-0-s001.docx]

**Barriers faced by surgeons in identifying and managing malnutrition in emergency general surgery: a qualitative study.**

Daniel L Ashmore^a,b^ BSc (Hons) MBChB (Hons) MRCS PGCert; dlashmore1@sheffield.ac.uk

Daniel M Baker^c^ BSc (Hons) MBChB (Hons) MRCS PGCert; bakerd989@gmail.com

Timothy R Wilson^b^ BSc (Hons) MBChB (Hons) FRCS PhD; tim.wilson1@nhs.net

Vanessa Halliday^a^ SFHEA, PhD, RD, RNutr (Public Health); vanessa.halliday@sheffield.ac.uk

Matthew J Lee^d^ BMedSci MBChB FRCS PhD; m.j.lee.1@bham.ac.uk

^a^School of Medicine and Population Health, Faculty of Health, University of Sheffield, Sheffield, UK

^b^Department of General Surgery, Doncaster and Bassetlaw Teaching Hospitals NHS Foundation Trust, Doncaster, UK

^c^Department of General Surgery, St James's University Hospital, Leeds Teaching Hospitals NHS Trust**,** Leeds, UK

^d^Institute of Applied Health Research, College of Medical and Dental Sciences, University of Birmingham, Birmingham, UK

*Corresponding author*

Mr Daniel L Ashmore

School of Medicine and Population Health, Faculty of Health, University of Sheffield, Beech Hill Road, Sheffield, UK, S10 2RX

dlashmore1@sheffield.ac.uk

ORCID ID 0000-0002-4321-5613; Twitter @realdanashmore

**Supplemental Material**

| **Supplementary Appendixes** |  |
| --- | --- |
| Interview schedule | *pag. 3* |
| **Supplementary Figures and Tables** |  |
| Table S1: Role and experience of each member of the research team | *pag. 6* |
| Table S2: Personal, interpersonal, methodological and contextual issues | *pag. 7* |

**Supplementary appendix – interview schedule**

**This is an outline for a semi-structured interview. This means that it adapts to the participant's response. The statements and questions below are intended as a guide to ensure relevant data is captured.**

Hello, my name is Daniel and I’m from the Medical School at the University of Sheffield. Thank you for agreeing to talk to me about how you identify malnutrition among NELA-eligible EGS patients, and what your experiences are of these.

If you’re still happy, I’ll record the conversation as planned. Please try to not use names during the interview. However, I would like to reassure you that anything you say will be anonymised and that it won’t be possible to identify you in any future publications or transcripts arising from this research. The digital recording will be transcribed and stored safely on the University of Sheffield’s systems until this is done. After this, the written transcription will only include a study number and the digital recording will be deleted.

If at any point you want to stop for a break, then please do speak up and of course, if at any point you want to stop and withdraw altogether, then again, please do let me know.

[State record study ID number and date of the interview]

1. **Tell me about yourself?**

Prompts:

- How many years as a consultant? How many years have you been doing EGS?
- What is your current hospital setting (IF unit, teaching, district)?
- Have you ever trained or worked in IF unit previously?
- Is there a Nutrition Support Team (NST) present in trust, and who makes up this team?

**The following questions are all in relation to NELA-eligible EGS patients.**

1. **How do you identify if a patient is at risk of malnutrition or are malnourished?**

Prompts:

- Do you use any nutrition screening or ‘assessment’ tools?
- Do you use any ‘nutrition markers’?
- Are there any other ‘global measures’? eg complexity of patient/ surgery/ likelihood of postoperative problems like ileus?
- Do you refer to the MUST score?
- Do you refer on to anyone like a dietitian?
- When do you first consider a patient’s nutrition status? (pre-/ post-operatively, and why?)

1. **Why is this?**

Prompts

- Personal factors – previous training
- Experience – have there been any specific cases that have shaped your decision making? To what extent does what you do (how you assess a patient) for a patient is shaped by past experience?
- Patient factors – unwell, access to GI tract
- Organisation factors – access to dietetic team? Does the process (ease of access) of delivering nutrition change their own practice in terms of how they identify malnutrition amongst EGS patients? Do local policies or protocols exist?
- External factors – are there any guidelines you use?

1. **Can you tell us what happens once a patient is identified as being at risk of malnutrition?**

Prompts:

- Is there a referral to a dietitian for a full assessment?
- Is there a reassessment? If so, at what stage? If not, why not?
- How does this change anything for the patient?
- Does it change when you operate? Or whom you operate on?
- Who makes the decisions regarding starting nutritional support in your trust?

1. **Specifically with regards to when you’re considering a patient’s nutritional status, are there any difficulties or barriers you encounter that prevent you from making the decision sooner than you do normally or would like to?**

Prompts:

- Why is this?
- Are there any issues with lack of time/ resources/ nutrition support team/ training/ team engagement?

Thank you for your time today, I really appreciate it.

1. **Is there anything you would like to add which hasn’t already been discussed?**
2. **Would being involved in further studies around this topic be something that interests you?**

**Supplemental Tables**

**Table S1:** Role and experience of each member of the research team.

| **Research team** | **Role and experience** |
| --- | --- |
| **DA** | - DA was the primary investigator. - DA is a PhD student and surgical trainee, and undertook all interviews. - DA has previous interview and qualitative research experience, which included using a structured approach to interview medical school applicants and formal training in Qualitative Research Design & Analysis. |
| **DB** | - DB is a surgical trainee - DB has coding and framework development experience. |
| **TW** | - TW is a consultant surgeon - TW has experience in mixed methods research. |
| **ML** | - ML is a consultant surgeon and clinical researcher. - ML has experience in mixed methods research. |
| **VH** | - VH is a registered dietitian and Faculty Director for Medicine, Dentistry and Health at the University of Sheffield. - VH has experience in mixed methods research. |

**Table S2:** Personal, interpersonal, methodological, and contextual issues.

| **Reflexivity orientation** | **Reflexivity processes** |
| --- | --- |
| **Personal** | - DA was the primary investigator and is a PhD student and surgical trainee. - DA has been senior surgical decision maker regarding which patients should be started on nutritional support for six years. - DA had trepidation regarding blind spots to the real underlying issues given his past experience. - DA’s experience may also lead to a degree of tacit knowledge which may impact the quality of the interview and subsequently to missing data. - All participants were aware of my dual roles and research interest. - Much of this research was conceived and driven by personal clinical experiences, and reinforced following my previous PhD work which showed a wide disparity among surgeons in approach to and management of malnutrition. |
| **Interpersonal** | - DA had previously worked with only 2/18 participants (P10 and P15). The recruitment strategy was developed to ensure participants were unlikely to have a pre-existing relationship. - To minimise power bias, these interviews arranged after DA had experience interviewing other participants and began to immerse himself in the data and early analysis. |
| **Methodological** | - Debriefing after interviews with the research team: The first three interview transcripts were reviewed by DA’s supervisory team in order to refine DA’s interview technique, aiming to limit tacit knowledge/‘shared surgical knowledge’ reducing the likelihood of missing data. - Immersion within the data through verbatim transcription by DA - Half of transcripts underwent dual review, independent coding and development of a thematic framework between DA and DB, with agreement from the research team. - Purposive recruitment of participants ensured an appropriate reflection of consultants regarding sex, years qualified, hospital setting and experience of intestinal failure. |
| **Contextual** | - Throughout recruitment, the research team were mindful of issues around surgical culture and diversity following the Kennedy report. Ensuring participants represented the surgical consultant body was important. |
